# Exploiting the high-resolution crystal structure of *Staphylococcus aureus *MenH to gain insight into enzyme activity

**DOI:** 10.1186/1472-6807-11-19

**Published:** 2011-04-22

**Authors:** Alice Dawson, Paul K Fyfe, Florian Gillet, William N Hunter

**Affiliations:** 1 Division of Biological Chemistry and Drug Discovery, College of Life Sciences, University of Dundee, Dundee, DD1 5EH, UK

## Abstract

**Background:**

MenH (2-succinyl-6-hydroxy-2,4-cyclohexadiene-1-carboxylate synthase) is a key enzyme in the biosynthesis of menaquinone, catalyzing an unusual 2,5-elimination of pyruvate from 2-succinyl-5-enolpyruvyl-6-hydroxy-3-cyclohexadiene-1-carboxylate.

**Results:**

The crystal structure of *Staphylococcus aureus *MenH has been determined at 2 Å resolution. In the absence of a complex to inform on aspects of specificity a model of the enzyme-substrate complex has been used in conjunction with previously published kinetic analyses, site-directed mutagenesis studies and comparisons with orthologues to investigate the structure and reactivity of MenH.

**Conclusions:**

The overall basic active site displays pronounced hydrophobic character on one side and these properties complement those of the substrate. A complex network of hydrogen bonds involving well-ordered water molecules serves to position key residues participating in the recognition of substrate and subsequent catalysis. We propose a proton shuttle mechanism, reliant on a catalytic triad consisting of Ser89, Asp216 and His243. The reaction is initiated by proton abstraction from the substrate by an activated Ser89. The propensity to form a conjugated system provides the driving force for pyruvate elimination. During the elimination, a methylene group is converted to a methyl and we judge it likely that His243 provides a proton, previously acquired from Ser89 for that reduction. A conformational change of the protonated His243 may be encouraged by the presence of an anionic intermediate in the active site.

## Background

Menaquinone, vitamin K_2_, is a lipid soluble quinone used by bacteria for electron transport. It is the sole quinone available to Gram-positive bacteria [1] whereas Gram-negative bacteria use ubiquinone under aerobic conditions, and menaquinone under anaerobic conditions [2]. Vitamin K, as menaquinone or the plant form phylloquinone, is a cofactor for γ-carboxylation of glutamate, a post-translational modification of proteins involved in blood coagulation and vascular biology and is an essential component of the mammalian diet [3].

There are two distinct biosynthetic routes to menaquinone. One, recently discovered, occurs in a small subset of bacteria such as *Helicobacter pylori *[4]. The starting point is chorismate supplied by the Shikimate pathway and the intermediates include the inosine derivative futalosine. The most prevalent, classical biosynthetic route to menaquinone, has been most studied in *Escherichia coli *[1]. The starting point remains chorismate, which is isomerized to isochorismate then converted, in three enzyme-catalyzed stages, to *o*-succinyl-1-benzoate. Two further reactions, involving ATP and CoA, lead to a CoA-naphthalene derivative before a thioesterase [5] removes the CoA moiety to release 1,4-dihydroxy-2-napthanoate. Prenylation, with products from the isoprenoid biosynthesis pathway, and finally a methylation reaction completes the production of menaquinone.

**Figure 1:**
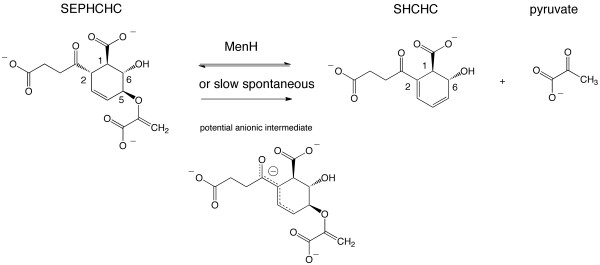
**The reaction catalyzed by MenH**. Figures 1 and 7 were prepared with Chemdraw (Adept Scientific).

To attain a comprehensive understanding of menaquinone biosynthesis, a fundamental aspect of bacterial metabolism, it is imperative to characterize the molecular structures and properties of the relevant enzymes. Our research in this area is confined to the classical menaquinone biosynthetic route and we previously reported on MenB [6] and MenD [7,8]. We turn our attention now to MenH, the enzyme which catalyzes the third step in this pathway, a 2,5-elimination of pyruvate from 2-succinyl-5-enolpyruvyl-6-hydroxy-3-cyclohexadiene-1-carboxylate (SEPHCHC) to produce 2-succinyl-6-hydroxy-2,4-cyclohexadiene-1-carboxylate (SHCHC, Figure 1).

There has been confusion about the reaction catalyzed by MenH, in large part due to misassignment of the enzymatic activities of MenB and MenD. Initial reports [9] described MenD as the enzyme that cleaved the pyruvate side chain from the isochorismate - α-ketoglutarate adduct SEPHCHC. MenB was assumed to cyclize and then cleave CoA from a napthanyl derivative [10], and the product encoded by the *menH *gene had no function assigned. It was subsequently proven that MenB lacked such CoA esterase activity and then postulated that MenH could catalyze this reaction [1]. Indeed, many databases still list *menH *as encoding a thioesterase. More recently, Jiang *et al. *[11] showed that MenD is not a bifunctional enzyme but is SEPHCHC synthase [E.C. 2.2.1.9]. Structural and kinetic studies are compatible with this assignment [7,8]. Rigorous biochemical analysis then proved that MenH is actually responsible for the elimination of the pyruvate side chain (E.C. 4.2.99.20) and that previous studies had been compromised by the inherent reactivity of SEPHCHC, which slowly decomposes to SHCHC and pyruvate [12].

We now describe the high-resolution crystal structure of *Staphylococcus aureus *MenH (*Sa*MenH), compare it to the structure of the *Vibrio cholerae *enzyme (*Vc*MenH) deposited in the Protein Data Bank (PDB) and discuss conserved residues with particular focus on what such residues contribute to the architecture of the active site. Consideration of data derived from site-directed mutagenesis and kinetic studies of the *E. coli *enzyme (*Ec*MenH) [12,13] allow us to reassess the molecular features that influence specificity and reactivity of MenH and address two issues of the structure-mechanism relationship, which remain unclear. The assignment of an oxyanion-binding site formed by two amino acid side chains by Jiang *et al. *[13] is highly unusual and is revisited. Finally the origin of the proton that converts a methylene group to a methyl (Figure 1) is considered and a proton-shuttle mechanism, involving transfer from substrate to protein then back at a different position is proposed.

## Results and Discussion

### General comments and overall structure

An efficient bacterial expression system for a histidine-tagged *Sa*MenH was constructed and supplied material for structural studies. Approximately 40 mg of protein was obtained from 1 L of *E. coli *culture. The enzyme crystallizes in the monoclinic space group *C*2 with one molecule in the asymmetric unit and diffraction data to better than 2 Å were measured in-house. The structure was solved by molecular replacement using a model based on PDB entry 1r3d, which although listed as a protein of unknown function from *V. cholerae*, has been identified as MenH [12]. The sequence identity with *Sa*MenH is about 20% and the best molecular replacement solution had a translational Z-score of 6.4, low for a correct solution. The initial electron and difference density maps were of poor quality but with defined features that suggested a correct molecular replacement solution had been obtained. Analysis of molecular packing in the crystal lattice also engendered confidence in the solution therefore we proceeded with refinement. The maps gradually improved with rounds of careful model building and refinement. Once all ordered MenH residues were included in the model a search for water molecules, to be included in the refinement, was initiated. A conservative approach to water identification was adopted. The crystallographic statistics and model geometry indicate that the refinement has produced an acceptable high-resolution model (Table 1). At the end of the refinement there was continuous electron density for the entire polypeptide chain, residues 1-266, missing only the Gly-His residues that remain at the N-terminus following proteolytic cleavage of the histidine tag.

**Table 1:** Crystallographic statistics.

Resolution range (Å)	19.73 - 1.94
Unit cell dimensions *a,b,c *(Å) *β *(°)	76.6, 43.6, 71.7, 98.3

Space group	*C*2

No. reflections measured, unique	71,206, 17.242

Multiplicity^a ^, Completeness (%)	4.1(3.3), 98.9 (93.3)

Wilson *B *(Å^2^)	24.5

*R*_*merge*_(%)^b ^, < I/?(I) >	5.3 (37.3), 17.5 (3.2)

Protein residues, water molecules	266, 185

*R*_*work*_(%)^c ^, *R*_*free*_(%)^d^	18.3, 24.1

DPI (Å)^e^	0.18

Average *B-factors *(Å^2^)	

Overall, main chain, side chain, waters	30.6, 29.3, 31.8, 31.3

R.m.s.d bond lengths (Å), angles (°)	0.012, 1.25

Ramachandran plot analysis (%)	

Favorable, Outliers	97.7, 0

^**a.**^Values in parentheses refer to the highest resolution shell. 2.05 - 1.94 Å ^**b. **^*R*_*merge *_= ∑_*hkl*_*∑*_*i*_*|*I_*i*_(*hkl*) - < I(hkl)>*|/ *∑_*hkl*_*∑*_*i *_I_*i*_(*hkl*); where I_*i*_(*hkl*) is the intensity of the *i*th measurement of reflection *hkl *and < I(hkl)> is the mean value of I_*i*_(*hkl*) for all *i *measurements. ^**c. **^*R*_*work *_= ∑_*hkl*_||*F*_*o*_|-|*F*_*c*_||/∑|*F*_*o*_|, where *F*_*o *_is the observed structure factor and *F*_*c *_is the calculated structure factor. ^**d. **^*R*_*free *_is the same as *R*_*cryst *_except calculated with a subset, 5%, of data that are excluded from refinement calculations.^**e. **^DPI, Diffraction-component Precision Index [39].

**Figure 2:**
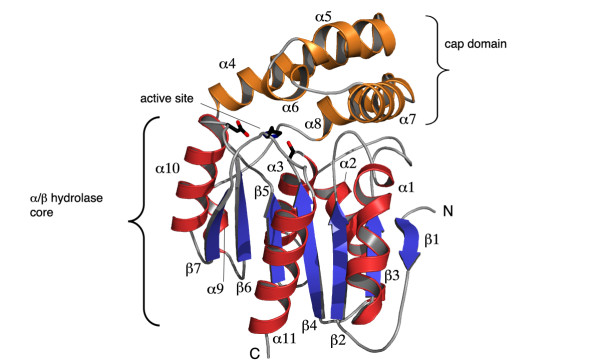
**The structure of *Sa*MenH and position of the active site**. The polypeptide is depicted as a ribbon with helices of the core domain colored red and b-strands blue, helices of the cap domain are copper colored. N and C mark the amino and carboxy termini of the protein. The catalytic triad consisting of Ser89, His243 and Asp216 are shown as sticks and mark the active site. Figures 2, 3, 4 and 6 prepared using PyMOL[41] and Illustrator (Adobe Systems Inc.).

Approximately 65% of the *Sa*MenH residues are in well-defined elements of secondary structure. These comprise 7 β-strands and 11 α-helices arranged in the α/β hydrolase fold [14,15], a central β-sheet with a marked twist, sandwiched between α-helices (Figure 2). Strand β1 is antiparallel to the others. Helices α1 and α11 lie in the concave face of the β-sheet and α4 to α8 form an insertion in the canonical α/β hydrolase fold to create a domain that caps the active site. The interface between the cap and core domains, which involves 11 hydrogen bonds, occludes approximately 25% of the surface area of the cap domain itself (~1500 Å^2 ^of ~5900 Å^2^).

Members of the α/β hydrolase family display different oligomerization states. For example, the C-C bond hydrolase MphC is dimeric in both solution and crystalline states, with an extended β-sheet formed across the dimer interface [16] whilst others such as acetylcholine esterase are monomeric [17]. *Sa*MenH behaves as a monomer in solution, as indicated by size-exclusion chromatography (data not shown). Analysis of the crystal structure using the Protein Interfaces, Surfaces and Assemblies server [18] indicated no significant areas of interaction to suggest oligomerization of MenH in the crystalline state (data not shown).

### Comparisons with related structures

There are over 660 structures known for α/β hydrolase family members and they encompass a wide range of differing functions such as esterases, hydrolases, lipases, haloperoxidases and peptidases [14,17,19]. The fold is tolerant to insertions, with proteins ranging from 200 to 600 residues in length. The canonical α/β hydrolase structure contains a core composed of eight β-strands [14,15]. However, in *Sa*MenH the core β-sheet consists of only seven strands due to the absence of the N-terminal strand observed in other family members. A common insertion in the canonical α/β hydrolase fold is a lid, or cap domain over the active site. In *Sa*MenH the cap is formed by five helices, α4 to α8 (Figure 2). The cap domain is important in defining the active site size and aspects of substrate specificity as will be described. In some structures the cap domain appears to be flexible as indicated by higher temperature factors. However, this is not the case in *Sa*MenH (data not shown) where we see no correlation between the selected Translation-Libration-Screw (TLS) domains and the cap/core domains. This observation should be treated with caution since the *Sa*MenH crystals contain a relatively low volume of bulk solvent (approximately 35%) and the molecular packing in the crystal lattice involves direct interactions of residues in the cap domain with three symmetry related molecules. The tight packing may therefore artificially restrict flexibility of the cap domain with respect to the core of the structure.

Analysis using Secondary Structure Matching [20] indicates that, as expected, *Sa*MenH is similar to various α/β hydrolases. However, with the exception of *Vc*MenH, there are no particularly close matches in the PDB. The structural homologues include haem-independent haloperoxidases, various esterases and hydrolases that overlay with *Sa*MenH with root-mean-squared deviation (r.m.s.d.) values in the range 2.0-2.4 Å, all with approximately 230 aligned Cα atoms and sequence identities around 20%. Similar results have been reported in structural comparisons of the α/β hydrolases, for example in an investigation of the carboxylesterase BioH from *E. coli *[21]. Of note is the Rv0554 protein from *Mycobacterium tuberculosis *recently investigated as a potential MenH orthologue, which falls within the range observed for the other α/β-hydrolases (r.m.s.d. 2.3Å for overlay of 227 Cα positions) and has no activity with SEPHCHC [22].

**Figure 3:**
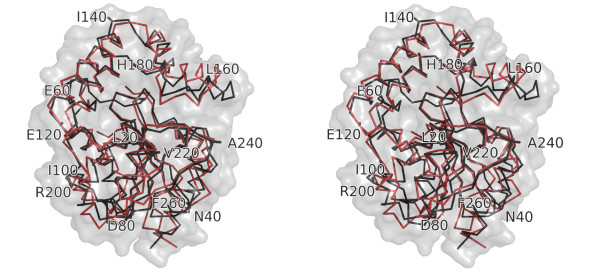
**Overlay of *Sa*MenH and *Vc*MenH**. A stereoview of the Cα trace of MenH from two species. *Sa*MenH is depicted as a semitransparent van der Waals surface (gray) with Ca trace as black sticks. The Ca trace of *Vc*MenH is shown as red sticks.

A closer alignment is observed when comparing *Sa*MenH with *Vc*MenH, proteins which share 21% sequence identity. The r.m.s.d. is 1.8 Å for least-squares fit of 232 Cα atoms (Figure 3). The loop linking β3 and α2, a region of high sequence variability in MenH orthologues (data not shown), is not modelled in the structure of *Vc*MenH but is ordered in *Sa*MenH. The largest difference between the two structures is in the orientation of the N-terminal region of β7, part of the lid domain, where Cα positions differ by up to 4 Å (Figure 3). This overlay highlights similarities in and around the active site that are likely important for enzyme activity and will be mentioned in the next section.

### The architecture of the MenH active site and recognition of substrate

**Figure 4:**
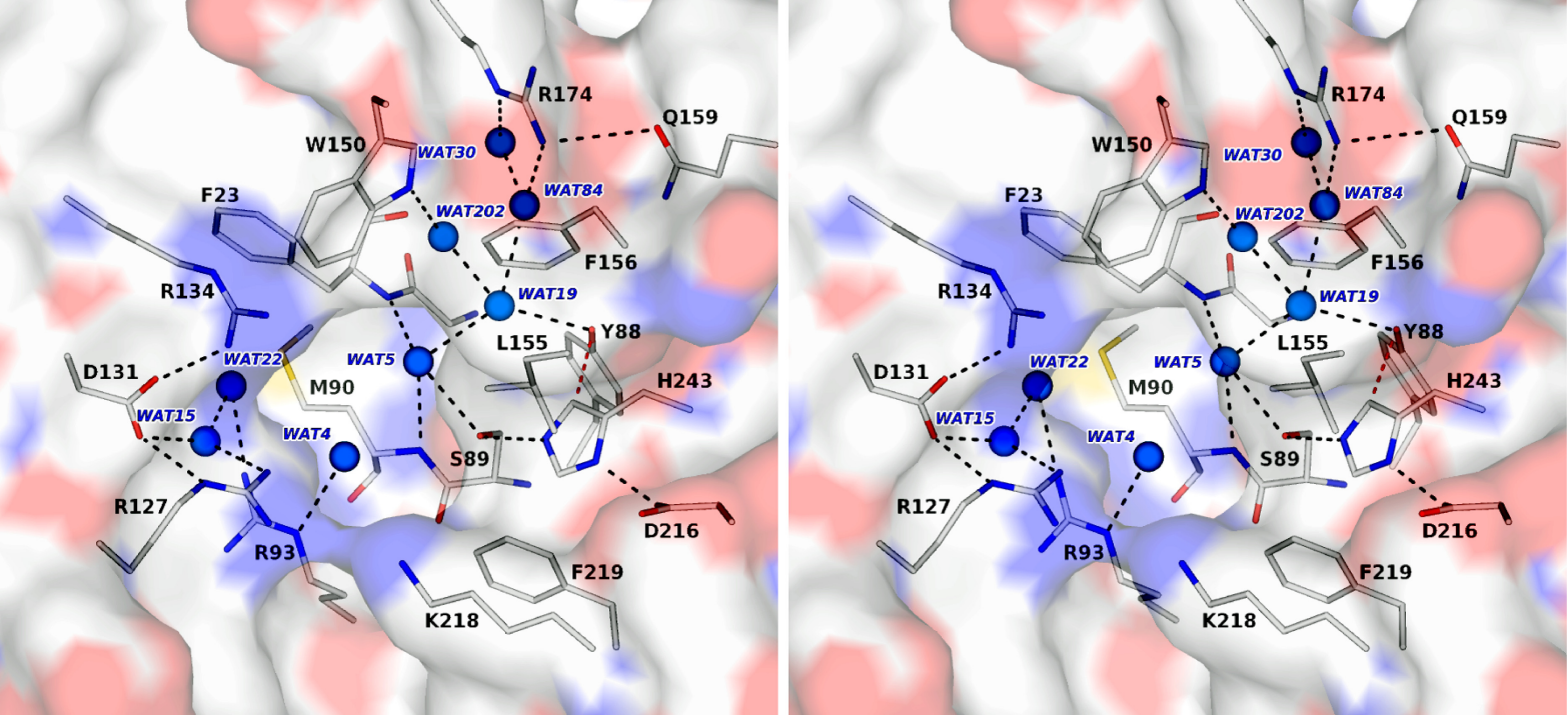
**Stereoview of the active site, catalytic triad and oxyanion-binding site**. *Sa*MenH is depicted as semi transparent van der Waals surface colored according to atom type (C grey, N blue, O red, S yellow). Selected amino acid side chains are shown as sticks colored according to atom type. The main chain of Met90 and Gly22-Phe23 are included to mark the oxyanion-binding site. Marine colored spheres represent selected water molecules. Black dashed lines mark potential hydrogen bonding interactions, the C-H•••O interaction between Tyr88 and His243 is represented by a dashed red line.

The residues that form the *Sa*MenH catalytic triad, Ser89, Asp216 and His243 are positioned on one side of a water-filled tunnel formed between the cap and core domains (Figure 4). The residues that contribute to the active site are contributed from α4, α5, α8, α10, two turns between β2-α1 and β4-α3 respectively and the loops that follow β5 and β7 (Figure 2). The entrance to the active site is near Arg127 and the tunnel then extends around and over the catalytic center to Asn32, a distance of about 25 Å (data not shown). A complex network of hydrogen bonds, involving well ordered water molecules, serve to organize the residues around the active site. The electrostatic properties complement the negatively charged substrate, which carries three carboxylate groups, at positions C1, C2, C5 and a hydrophobic component (Figure 1). A cavity is formed to the side of this tunnel near Phe23, adjacent to the catalytic machinery (Figure 4).

**Figure 5:**
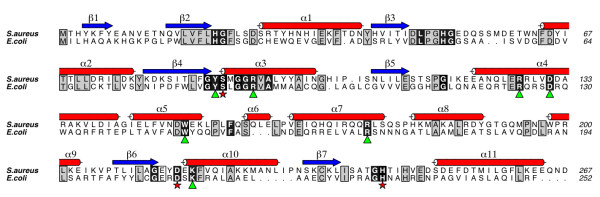
**A sequence alignment of *Sa*MenH and *Ec*MenH**. Residues outlined in black are 90% conserved in 101 MenH sequences. Residues outlined in grey are conserved in *Sa*MenH and *Ec*MenH. Residues marked with a star form the catalytic triad and those residues were mutated by Jiang *et al.*[13] in *Ec*MenH are marked with a triangle. This figure was prepared using MUSCLE [42] and ALINE [43].

As is typical of the α/β hydrolase fold, the catalytic serine (Ser89) occurs in a GXSXG motif (Figure 5) located on a tight turn, often termed the 'nucleophile elbow', between a central β-strand and a helix. The two glycine residues help create the tight turn, which is also stabilized by a hydrogen bond formed between the main chain carbonyl of Ser89 and amide nitrogen of Gly92 (data not shown). Asp216 is located on a second tight turn, between β6 and α10, and is shielded from the active site by Leu155 and Phe219. The hydrogen bonding interaction between Asp216 and His243, helps to position the imidazole to interact with the hydroxyl of Ser89 in the apo-structure. Ser89 OG also accepts a hydrogen bond donated from a particularly well-ordered water molecule (*B *= 7 Å^2^, where *B *is the Debye-Waller or isotropic temperature factor, 8π^2^〈*u*^2^〉 with *u *the atomic displacement parameter), which in turn accepts two hydrogen bonds from the main chain amides of Phe23 and Met90.

The active site has a pronounced hydrophobic patch created by Trp150, Phe156, Leu153, Leu155 and Phe219, and in addition Phe23 and Leu186 (not shown) help form the base of the site (Figure 4). The position of Trp150 is determined by van der Waals interactions with Phe23 and Phe146 (not shown) on one side of the indole, Phe156 on the other side and the side chain of Arg134 abuts CZ3. This orientation of the Trp150 side chain places NE1 directed towards the active site to form a hydrogen bond with an ordered water molecule (Figure 4) that contributes to forming a network of solvent molecules in the active site. Phe219, Leu155 and Phe156 provide a hydrophobic environment around the side chain of His243 helping to hold it in place and providing a microenvironment that may increase the p*K*_a_.

The side chain of Tyr88 is positioned by van der Waals interactions with Gly22 Cα, Ser89 Cβ, Glu112 CD and His246 CE1. His246 is held in place by hydrogen bonds formed between ND1 and NE2 with Glu112 OE1 and Thr29 OG1 respectively (data not shown). As a result, Tyr88 OH is placed to participate in hydrogen bonds with Thr244 OG1 and an ordered water molecule, (*B *= 18 Å^2^). A distance of 3.2 Å separates His243 CD2 from Tyr88 OH and the geometry is compatible with the presence of a C-H•••O hydrogen bond (Figure 4) [23]. The C-H•••O hydrogen bond is a weak interaction but may contribute stability to the positive-charge distribution on the protonated histidine and in so doing support a catalytic function. Such a role has been invoked in enzymes such as trypanothione reductase, which is dependent on proton transfer from histidine [24].

There are six basic residues in the active site (Arg93, Arg127, Arg134, Arg174, Lys218 and the catalytic His243) and a single acidic residue, Asp131 (Figure 4). Lys218 is on the periphery of the active site with the aliphatic component of the side chain lying on the side chain of Phe219. Arg127 and Arg134, together with Asp131 are opposite Lys218. Arg127 forms both a direct and a water-mediated hydrogen-bonding association with Asp131, which also accepts a hydrogen bond from Arg134. The water that bridges Arg127 and Asp131, Wat15 (*B *= 17 Å^2^) accepts a hydrogen bond donated by the amide of Thr191. Arg93 is held in place by forming a hydrogen bond to the main chain carbonyl of Tyr189 and to two water molecules in the active site, Wat22 (*B *= 21 Å^2^) and Wat4 (*B *= 22 Å^2^). Arg174 is about 10 Å from the catalytic center lining the polar tunnel formed at the interface of the cap and core domains. Arg174 NH2 donates a hydrogen bond to Gln159 OE1 and to four water molecules, two of which are shown in Figure 4.

The overall sequence identity between MenH proteins is relatively low and a comparison highlights key residues in and around the active site (Figure 5). Of 256 orthologues in the Kyoto Encyclopedia of Genes and Genomes (KEGG) [25], 101 are unique when filtered at a 90% sequence identity level. Within these sequences, 23 residues are conserved in at least 90% of them. The catalytic triad is conserved in all but one sequence where aspartate is replaced by glutamate. Eight of the remaining strictly conserved residues are glycine, which are all located at the end of secondary structure elements or in loops and may be important in defining the overall structure, for example in the "elbow" motif in the active site. The acidic Asp131, five of the nine hydrophobic residues and five out of the six basic residues listed above as contributing to the architecture of the active site are strictly conserved. Three of the hydrophobic residues, Met90, Phe146, Leu155 are replaced by valines or leucine. Arg134 is not a highly conserved residue, being substituted by Trp131 in *Ec*MenH for example (Figure 5).

The structural overlay of *Sa*MenH and *Vc*MenH, discussed above, indicates that the conserved residues in the active site adopt similar orientations and only the side chain of Arg174 (*Sa*MenH numbering) differs significantly (data not shown). In *Sa*MenH, Arg174 is oriented towards α5. In *Vc*MenH, the corresponding residue, Arg176, adopts a different rotamer and the side chain is directed towards α1 whilst Tyr156 occupies the site of the *Sa*MenH Arg174 guanidinium (data not shown). This observation suggests a degree of conformational freedom deep in the active site that might influence the position of the cap domain.

**Figure 6:**
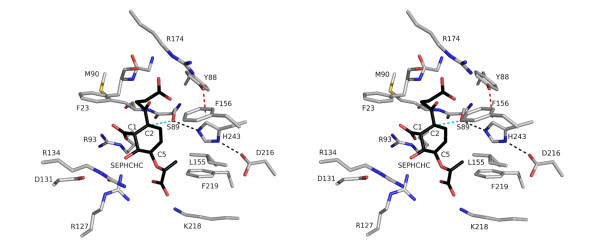
**A model for substrate binding to *Sa*MenH**. A stereoview of the ES complex model. SEPHCHC is depicted with C positions colored black. MenH residues are shown as in Figure 4. Trp150 is not shown for the purpose of clarity. The chemical structure modelled represents Stage I in Figure 7.

Attempts to soak ligands such as pyruvate, salicylic acid and the substrate SEPHCHC into crystals of *Sa*MenH, and so derive structures directly relevant to substrate recognition resulted in the loss of diffraction. Co-crystallization experiments were also carried out but either the crystals did not grow or when available and subsequently analyzed, there was no electron density that could be attributed to these ligands (data not shown). In the absence of any experimentally determined structures we sought to prepare a simple model of the enzyme-substrate (ES) complex. We were guided by seeking a position of SEPHCH in the active site in which there was little steric hindrance and where the chemical properties of substrate and active site were complementary to each other. The potential for His243 to act as a base and acquire the C2 proton direct from substrate was recognized. However, when the substrate was positioned to facilitate the interaction of SEPHCH C2 with His243 NE2 there were severe steric clashes with the protein. The positions of the Leu155 and Phe156 side chains in particular appear to compromise such a model. We cannot rule out the possibility that gross structural changes might change the active site to facilitate the use of His243 as an active site base for the MenH reaction but note that there is no evidence to support such conformational changes to the catalytic residues utilized within the α/β hydrolase family of enzymes. An alternative model was constructed by placing SEPHCHC C2 3.3 Å from Ser89 OG. The ligand was simply rotated about C2 to minimize steric clash with the enzyme (Figure 6) and, as will be described, the model suggests plausible chemical interactions to stabilize the substrate in the enzyme active site.

Our ES model provides a template to consider aspects of substrate recognition and MenH catalysis. In this we expect that important features relevant to substrate recognition and catalysis be conserved in MenH sequences and the α/β-hydrolase fold as discussed above. We noted also that the position of water molecules might identify where functional groups of substrate could bind. A thorough analysis, combining kinetic studies with the use of site-directed mutagenesis studies has been applied to seven conserved MenH residues using the *E. coli *enzyme (Figure 5). The kinetic results were interpreted in light of a homology model of *Ec*MenH based on *Vc*MenH combined with the use of docking calculations [12,13]. Our *Sa*MenH ES model (Figure 6), which should be consistent with the kinetic data, indeed corroborates certain aspects of the previous study but there are significant differences in the models, which we will detail.

Our ES model suggests that Arg127 is placed to interact with the C6 hydroxyl and or the C5 carboxylate. Lys218 could also interact with the C5 carboxylate and Arg93 is well placed to form a salt-bridge interaction with the C1 carboxylate. The substrate C2 carboxylate, the succinyl substituent, is positioned in the solvent filled channel and placed to accept hydrogen bonds donated from Tyr88 OH and Trp150 NE1. The hydrophobic component, C3, C4 and the methylene group are positioned over towards the hydrophobic patch on the active site near Leu155 and Phe156. Mutations of the conserved Arg93, Arg127 and Arg174 to alanine leads to increased *K*_M _values and reduced catalytic properties. This is compatible with a role of these arginine residues in binding and orientating the substrate in the active site. Arg174 does not interact with substrate in the ES model but we cannot rule out a conformational change that facilitates an interaction between the guanidinium and the C2 carboxylate. The side chain of Arg174 is implicated in positioning Trp150 and disruption of the structure in this part of the active site might contribute to the compromised level of enzyme activity. Asp131 interacts with Arg127 and mutation of the corresponding residue, Asp128, in *Ec*MenH to alanine leads to an eight-fold increase in *K*_M _[13] consistent with localized disruption to substrate binding.

The mechanism of typical α/β hydrolases involves the formation of an oxyanion intermediate stabilized in the active site by an "oxyanion-binding hole" usually formed by two main chain amides. One amide is contributed from the residue immediately following the catalytic serine, the second can vary in location but often resides on the loop linking a β-strand and helix. Jiang *et al. *[13] proposed that the oxyanion hole is located between the side chains of Tyr88 and Trp150 (*Sa*MenH numbering) based on results of computational docking calculations. This would be an extremely unusual situation not previously observed in this enzyme family. In *Sa*MenH the main chain amides of Met90 and Phe23 are well placed to form the oxyanion hole; indeed a well-ordered water molecule is observed within hydrogen bonding distance of both nitrogen atoms in addition to the side chain of Ser89 (Figure 4). A similar situation occurs in *Vc*MenH, with the corresponding residues being Ser91, Leu92 and Leu24. Structural alignment with other α/β hydrolases where the oxyanion hole has been identified support our interpretation [26,27]. In our ES model, a carbonyl oxygen of the succinyl substituent, where a negative charge might build up, overlays on the position of this ordered water molecule (Figure 6). That being the case we suggest a different role for the conserved tryptophan (*Sa*MenH Trp150). Mutation of the equivalent residue in *Ec*MenH, Trp147 to phenylalanine, had significantly less effect on catalysis than a Trp147 to alanine mutation [13]. This further suggests that the side chain is not actually involved in the formation of the oxyanion hole. Our ES model suggests that Trp150 stabilizes the hydrophobic part of the active site by interactions with Phe146, Phe156 and Leu153 and that Trp150 NE1, in partnership with Tyr88 OH, donate hydrogen bonds to bind the carboxylate of the succinyl side group of the substrate (Figure 6). Alteration of Tyr88 to phenylalanine increases *K*_M _slightly but reduces catalytic efficiency to a marked degree. This would support the earlier proposal that interaction with His243 might influence catalysis.

### A proposed mechanism for MenH

All α/β hydrolases contain a catalytic triad consisting of a nucleophile, an acid and a histidine. In many cases, the reaction proceeds in a similar fashion to that of the serine proteases, where the substrate is subject to nucleophilic attack by serine, for example prolyl oligopeptidase [28]. The histidine and aspartate components of the catalytic triad are essential for *Ec*MenH activity; mutation of the serine resulted in a considerably less active, but still functional enzyme, suggesting a mechanism distinct from most other α/β hydrolases [12,13] but similar to the C-C bond hydrolases MhpC and BhpD. Mutation of the active site serine in MhpC reduces but does not abolish enzyme activity and additional kinetic studies lead to the assignment of a general base catalysis mechanism [29]. The non-essentiality of the active site serine in MenH suggests that it may also adopt a general base mechanism.

**Figure 7:**
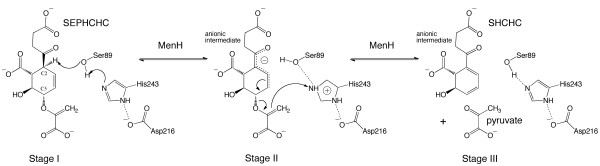
**A proposed mechanism for MenH**.

The reaction catalyzed by MenH can be described in three stages (Figure 7). In stage I, the catalytic triad structure positions His243 to abstract a proton from Ser89 OG and so prepare a seryl oxide which in turn supports removal of the acidic C2 proton from SEPHCHC. An anionic intermediate is generated. Stage II is breaking the C5-O bond to release the pyruvate side chain; a common step in biosynthetic reactions that use chorismate. However, in most cases, the product is aromatic [30] and MenH is unusual in generating a partially saturated product. Nevertheless, the stability expected when a conjugated system is present is likely a major driving force in pyruvate elimination from C5 subsequent to the abstraction of the proton from C2. In stage III, methylene is reduced to the pyruvate methyl by addition of a proton and the product then leaves the active site.

Whereas mutation of the active site serine does not completely destroy MenH activity, alteration of the catalytic His243 is catastrophic. The explanation may reside in the combination of two factors. Firstly, the substrate has a relatively labile acidic C2 proton irrespective of whether a seryl oxide is nearby to acquire it or not. The difference is in the rate of proton loss. Secondly, His243 is primed by association with Asp216 and Tyr88 to abstract a proton from any nearby hydroxyl group or a water molecule, which can then be passed on to reduce the methylene to a methyl. An attraction between this intermediate and the protonated His243 could assist a movement of the amino acid side chain into a position whereby donation of a proton to the methylene group is possible.

## Conclusions

The high-resolution crystal structure of *Sa*MenH, an α/β hydrolase, has been determined and detailed comparisons carried out with proteins of related fold. The MenH active site properties complement those of the substrate, which is negatively charged and possesses significant hydrophobic character. The enzyme:substrate complex has been modelled and a potential mechanism, exploiting previously published data combining site-directed mutagenesis with kinetic studies of *Ec*MenH, is proposed. The reaction may be initiated by proton abstraction from the substrate by an activated Ser89, part of a catalytic triad with Asp216 and His243. The driving force for pyruvate elimination is provided by the formation of a conjugated system. During pyruvate elimination, a methylene group is converted to a methyl and it is possible that His243 provides a proton, acquired from Ser89 for that reduction.

## Methods

### Expression and purification of *Sa*MenH

Chemicals, of the highest quality available, were sourced from Sigma-Aldrich and VWR International except where stated otherwise. The *menH *gene was amplified by PCR from *S. aureus *genomic DNA (strain American Type Culture Collection 35556) using 5'- **CATATG**ACACACTACAAGTTTTATGAAGC-3' as the forward primer and 5' - **GGATCC**TTAGTCATTTTGCTCCTCC - 3' as the reverse primer (Thermo Scientific). These oligonucleotides contain 5' *Nde*I and 3' *Bam*HI sites indicated in bold. The PCR product was ligated into TOPO-BLUNT-II (Invitrogen) and then subcloned into a modified pET15b vector (Novagen), which produces an N-terminally hexa-histidine tagged protein with a Tobacco Etch Virus (TEV) protease site. The resulting expression vector was heat shock transformed into *E. coli *strain BL21 (DE3) (Stratagene). Cell cultures were grown in 1 L volumes using Luria-Bertani media supplemented with 50 μg mL^-1 ^of carbenicillin at 37°C to an optical density of 0.6. The cultures were cooled to room temperature, expression induced using 0.5 mM isopropyl-β-D-thiogalactopyranoside, and growth continued for 16 hours at room temperature.

Cells were harvested by centrifugation for 25 minutes at 40,000 g at 4°C, resuspended in lysis buffer (50 mM Tris-HCl, 250 mM NaCl, 20 mM imidazole pH 7.5) containing DNase I (0.1 mg), and a single tablet of a cocktail of EDTA-free protease inhibitors (Roche), and lysed using a French press at 1000 psi. Insoluble debris was separated by centrifugation at 39,000 g for 25 minutes, at 4°C. The supernatant was loaded onto a 5 mL HisTrap HP column (GE) precharged with Ni^2+^. A linear concentration gradient of imidazole was applied, with *Sa*MenH eluting at approximately 160 mM imidazole. Fractions were analyzed using sodium dodecyl sulfate polyacrylamide gel electrophoresis (SDS-PAGE), and those containing *Sa*MenH were pooled and incubated with TEV protease at 30°C for three hours. The untagged protein was separated from the TEV protease, tag and residual tagged protein using a second Ni^2+ ^charged HisTrap column; the untagged protein still bound weakly to the column, eluting at approximately 80 mM imidazole. The untagged *Sa*MenH was then further purified using a Superdex 200 26/60 gel filtration column (GE), equilibrated using 50 mM Tris-HCl, 250 mM NaCl pH 7.5. The protein eluted as a single species with mass of approximately 30 kDa consistent with that of a monomer. Selected fractions were pooled, dialyzed into 20 mM Tris-HCl, 50 mM NaCl, pH 7.5 and concentrated for crystallization. The protein concentration was estimated spectrophotometrically using a theoretical extinction coefficient of 32,890 M^-1 ^cm^-1 ^(ProtParam) [31].

### Crystallization and data collection

Initial screening was performed using sitting drop vapor diffusion with standard sparse matrix screens. Multiple hits were obtained in conditions containing polyethylene glycol (PEG). Further tests with a PEG screen (Qiagen) yielded a diffraction quality crystal from reservoir conditions 0.1 M sodium HEPES pH 7.5, 25% w/v PEG 4000 in a drop consisting of 1 μL of protein solution (5 mg mL^-1 ^) and 1 μL of reservoir. Rod shaped crystals took several weeks to appear at 20°C. A crystal (approximately 0.5 × 0.08 × 0.08 mm^3^) was mounted directly from the drop, without any additional cryoprotection, into a flow of cooled nitrogen at -173°C. Data were collected in-house using a Rigaku Micromax 007 rotating anode (Cu Kα λ = 1.5418 Å) R-AXIS IV^++ ^image plate system. The data were integrated using XDS [32] and scaled with Scala [33]. Statistics are presented in Table 1.

### Structure solution and refinement

The search model for molecular replacement calculations was derived from PDB entry 1r3d, the MenH orthologue from *V. cholerae*. Non-conserved side chains were truncated to alanine using Chainsaw [34], and molecular replacement carried out in Phaser [35]. Initial rigid body refinement gave an R-factor of 55%. Rounds of model manipulation using Coot [36] interspersed with refinement using Refmac5 [37] were used to complete the protein model; addition of water molecules then completed the refinement. The *R*_work _and *R*_free _values converged at 18.3 and 24.1% respectively. TLS refinement using three domains (analysis used the TLS server [38]) was included as part of the refinement. The three domains consisted of residues 2-45, 46-119 and 120-265. The Diffraction-component Precision Index (DPI), as defined by Cruickshank [39], and the Ramachandran plot statistics, as calculated in Molprobity [40], are given in Table 1.

### Protein Data Bank accession numbers

Coordinates and structure factors have been deposited with accession code 2xmz.

## Abbreviations

CoA: coenzyme A; E.C.: Enzyme Commission; PEG: polyethylene glycol; PDB: Protein Data Bank; r.m.s.d.: root mean square deviation; *Sa*: *Staphylococcus aureus*; SEPHCHC: 2-succinyl-5-enolpyruvyl-6-hydroxy-3-cyclohexadiene-1-carboxylate; SHCHC: 2-succinyl-6-hydroxy-2,4-cyclohexadiene-1-carboxylate; TEV: Tobacco Etch Virus; TLS: Translation/Libration/Screw; *Vc*: *Vibrio cholerae*.

## Authors' contributions

AD cloned, expressed, purified and crystallized *Sa*MenH, refined the structure and drafted the manuscript. PKF collected data and aided in the refinement. FG aided in protein purification and crystallization. WNH conceived of the study and drafted the manuscript. All authors read and approved the final manuscript.
